# *Phylo.io*: Interactive Viewing and Comparison of Large Phylogenetic Trees on the Web

**DOI:** 10.1093/molbev/msw080

**Published:** 2016-04-19

**Authors:** Oscar Robinson, David Dylus, Christophe Dessimoz

**Affiliations:** ^1^Department of Computer Science, University College London, London, United Kingdom; ^2^Department of Genetics Evolution and Environment, University College London, London, United Kingdom; ^3^Department of Ecology and Evolution, University of Lausanne, Lausanne, Switzerland; ^4^Center for Integrative Genomics, University of Lausanne, Lausanne, Switzerland; ^5^Swiss Institute of Bioinformatics, Lausanne, Switzerland

**Keywords:** Phylogenetics, visualization, web services, molecular evolution.

## Abstract

Phylogenetic trees are pervasively used to depict evolutionary relationships. Increasingly, researchers need to visualize large trees and compare multiple large trees inferred for the same set of taxa (reflecting uncertainty in the tree inference or genuine discordance among the loci analyzed). Existing tree visualization tools are however not well suited to these tasks. In particular, side-by-side comparison of trees can prove challenging beyond a few dozen taxa. Here, we introduce *Phylo.io*, a web application to visualize and compare phylogenetic trees side-by-side. Its distinctive features are: highlighting of similarities and differences between two trees, automatic identification of the best matching rooting and leaf order, scalability to large trees, high usability, multiplatform support via standard HTML5 implementation, and possibility to store and share visualizations. The tool can be freely accessed at http://phylo.io and can easily be embedded in other web servers. The code for the associated JavaScript library is available at https://github.com/DessimozLab/phylo-io under an MIT open source license.

Phylogenetic analyses often require the inference and evaluation of multiple trees for the same group of taxa—either to gauge the uncertainty in the inference (e.g., by sampling trees from the posterior distribution) or to observe incongruence among different loci (e.g., resulting from horizontal gene transfer). Therefore, there is an increasing need for tools that facilitate quantitative and qualitative comparison of phylogenetic trees. In particular, the need for visualization of large trees (>500 taxa) has driven the development of many different approaches, some of which are not only related to applications in biology (reviewed in von Landesberger et al. 2011).

Popular tools for tree visualizations are FigTree (Rambaut 2009) and EvolView (Zhang et al. 2012). FigTree allows users to display and manipulate tree visualizations in detail. Written in Java, it is a platform-independent standalone tool. Multiple trees can be loaded simultaneously and it is possible to browse through each individual tree. EvolView, on the other hand, is accessible through a web interface. EvolView allows users to map this information onto the tree visualization, thus providing the user with an understanding of evolutionary events on the genome level. While both tools are well suited to analyze single trees, there is no function to compare different topologies. Moreover, visualization of large trees can become cumbersome. An additional tool to visualize phylogenetic trees is part of a bigger software package, that is, the molecular evolutionary genetics analysis package (Tamura et al. 2013). This tool allows to display basic newick trees and to manipulate a single tree. However, similar to other tools, comparison of different trees is not supported.

Phylogenetic network visualization tools such as SplitsTree (Huson and Bryant 2006) and DensiTree (Bouckaert 2010) display multiple trees at once. SplitsTree represents trees as a network and DensiTree overlays trees to create a composite image. However, the aggregated resulting visualizations make it difficult to pinpoint the specific changes between two trees. Furthermore, when the size of the trees increases, the legibility of the visualizations rapidly degrades.

A common method for side-by-side tree comparison is the use of “tanglegrams” (Scornavacca et al. 2011). In this approach, leaves corresponding to the same species are linked with lines, with the dissimilarity between the two trees reflected in the number of line crosses. However, tanglegrams are difficult to read and interpret when there are substantial differences between two trees, and they too scale poorly to large trees.

For direct tree comparison, the best tools rely on color coding and visual node annotation. Compare2Trees (Nye et al. 2006) annotates nodes using a novel algorithm for tree structure comparison. However, it does not scale well when large trees are compared. Although not specifically developed to visualize phylogenetic trees, TreeVersity (Gomez et al. 2012) is another tool that facilitates detailed visual comparison of trees, but is again limited in terms of its scalability. Another tool for side-by-side comparison is TreeJuxtaposer (Munzner et al. 2003). It is scalable for large trees and enables users to compare subtrees. Unfortunately, it has not been maintained for a long time and is cumbersome to start because it is not up-to-date with current Java security standards.

The final drawback with some current state-of-the-art tools is their availability. Compare2Trees, a web-based tool, for example, is build as Java Applet and therefore difficult to use on modern web browsers. Desktop software, on the other hand, may require legacy systems or only run on specific platforms. Significant contributions such as PhyloComp (Bremm et al. 2011) are, unfortunately, not readily available for download and installation. Moreover, PhyloComp also suffers from the same visual and computation scalability issues as many other tools. However, software facilitating phylogenetic tree visualization in modern browsers is beginning to appear. One example is the jsPhyloSVG library (Smits and Ouverney 2010). This library enables users to visualize and manipulate single trees. However, similarly to most other tools, it does not offer any function for comparing trees.

In summary, we can identify three main drawbacks with current tools: (1) a lack of functionality to compare trees; (2) a failure to scale beyond a few dozen taxa; and/or (3) the use of outdated technology, thereby compromising their usability.

Here, we present *Phylo.io*, a web-based software for tree viewing and side-by-side comparison. Similarities and differences between two trees are indicated with a color scheme, and it is possible to highlight corresponding nodes and clades of interest between trees. The tool is scalable to large trees while maintaining tree legibility and quantitative comparison of the tree structures. Moreover, it is able to find the best corresponding rooting and order of leaves between two trees, facilitating their side-by-side comparison. Finally, the tool supports saving and sharing of a current state of visualization via custom URLs, thereby facilitating collaborative work on large trees.

## New Approach

*Phylo.io* is built using HTML5, CSS, Ajax, jQuery, and the D3 JavaScript visualization library (Bostock et al. 2011). Therefore, it does not need to be installed and is instantly accessible and usable on all modern web browsers. In addition, all computations are performed on client side, making it inherently scalable.

In order to perform a tree analysis, the user can choose between two modes using the newick format as input. First, the “view” mode makes it possible to display a single tree. There, basic tree operations can be performed directly on the nodes and branches of the tree, that is, re-rooting and branch swapping. Second, with the “compare” mode, two trees are displayed side-by-side. The differences and similarities of both trees are highlighted on the branches and nodes using a ColorBrewer color scheme (Harrower and Brewer 2003). The degree of similarity, indicated by a color scale, is calculated using a variation of the Jaccard index that is optimized for speed, as presented in Munzner et al. (2003) and Bremm et al. (2011). All basic tree operations remain possible in this mode.

*Phylo.io* improves the legibility of large trees by estimating an optimal collapsing depth using the number of leaves and size of viewing area and displaying automatically a collapsed version of the tree. The underlying data structure stores tree nodes in an object that contains two lists, one with the visible nodes and the other with nodes that are collapsed. Therefore, if the rendering function reaches a node with collapsed subtree, it simply stops to render, thus making the interface more responsive. This also ensures that all data remain available for analysis but are not shown until required. During a first rendering pass, nodes beyond a certain depth (automatically estimated from the available screen size) are collapsed into a composite node that is represented by a triangle. The initial collapsing provides two benefits: (1) the tree remains legible, as branches are not bunched up and text is not overlapping; and (2) the rendering is fast because not all subtree comparisons have to be calculated. Therefore, smaller trees are rendered instantaneously and larger trees in a relatively short time (for a tree with 500 taxa, a few seconds on a laptop). Furthermore, the tool provides a search functionality that colors the branches from a root to a queried leaf. In the “compare” mode, as branches are expanded, the best corresponding node in the opposing tree for each node in each newly visible branch is calculated. Therefore, the computationally expensive task of calculating the score and location of the best corresponding node is split up and only calculated just before the rendering. This enables the tool to remain as responsive as possible.

When comparing two trees, a common task is to match leaves in the tree or to find the best corresponding internal node in the opposing tree. In *Phylo.io*, the user can compute the best corresponding rooting. Moreover, an automated branch-swapping procedure allows users to find the best corresponding visualization between two trees. This is useful in a broad range of contexts, such as to compare trees that have been inferred using different methods, to compare samples from a Bayesian posterior distribution, or to compare differences in the trees reconstructed from different loci.

For interactive analysis of specific parts of the tree, the user can select a node and highlight it. The best corresponding node in the opposing tree is then highlighted and centered, allowing the user to interactively match structures within the compared trees. If the best corresponding node is in a collapsed subtree, all nodes on the path to that node will be expanded making the node visible. Therefore, regardless of the current collapsing depth, the user can always find the corresponding structure in the opposing tree without having to view the entire tree.

Finally, *Phylo.io* allows users to share tree visualizations using the GitHub Gist API (which supports storage and retrieval of data). *Phylo.io* stores the current tree data structure in an extended newick format, where information about the visualization are preserved as metadata and thus save the current visualization state. The share functionality generates a unique URL that can be shared with collaborators, who in turn can retrieve and work on the tree in its current state.

**Fig. 1:**
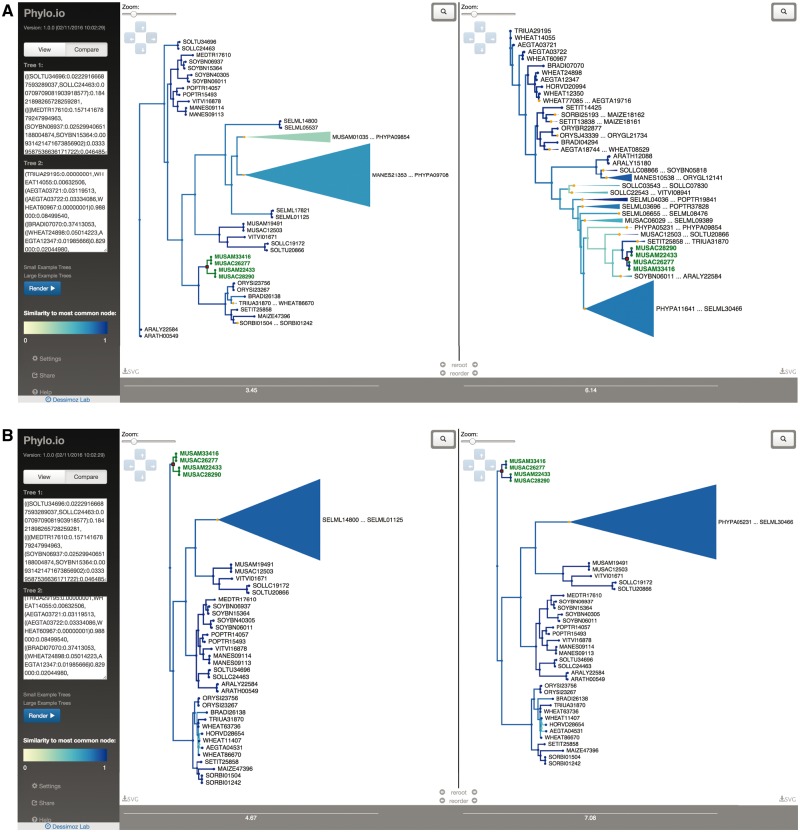
*Phylo.io* web interface in “compare” mode. (*A*) Two large trees with the same 737 leaves but different topologies in compare mode. The yellow to blue color scheme indicates the similarity of best matching subtrees between the two trees. In the left tree, an inner node (highlighted in red) is selected, thereby highlighting its subtree (in green) and corresponding parts in the right tree (in green). (*B*) The same tree with the same highlighted inner node, but after automatically re-rooting and reordering subtrees.

To illustrate the usefulness of *Phylo.io*, two trees generated using different methods, one using PhyML (Guindon et al. 2010) and one using RAxML (Stamatakis 2014) were computed on data retrieved from the OMA Orthology Database (Altenhoff et al. 2015)—specifically the Hierarchical group HOG:0152954.2aq.7d at Embryophyta level. These trees contain 737 proteins and are viewable in the application as the “Large Example Trees” and are displayed in figure 1. In this example, although the two programs return seemingly very different trees in terms of their Newick strings, by automatically re-rooting and re-ordering subtrees for maximum consistency between the two trees, *Phylo.io* makes it obvious that the (un-rooted) topologies returned by the programs are, in fact, near identical.

*Phylo.io* can also be used as embedded tree viewer in other resources. For instance, the MAFFT aligner online server (Katoh and Standley 2013) and aLeaves resource for homolog sequence search (Kuraku et al. 2013) use *Phylo.io* to display trees. Instructions to do so are available as part of the source code documentation.

In conclusion, *Phylo.io* addresses the need for a usable and scalable tree viewer, with particularly useful features for side-by-side tree comparison. However, as with any new tool, there is a long list of future functionality ideas. For instance, we would like to support other types of input formats and extend side-by-side comparison to trees with partially overlapping leaf sets. Meanwhile, by releasing *Phylo.io* under a permissive open source license, we also encourage improvements and bug fixes by the broader community.
